# *BpGRP1* acts downstream of *BpmiR396c*/*BpGRF3* to confer salt tolerance in *Betula platyphylla*

**DOI:** 10.1111/pbi.14173

**Published:** 2023-09-13

**Authors:** Zhongyuan Liu, Tengqian Zhang, Ruiting Xu, Baichao Liu, Yating Han, Wenfang Dong, Qingjun Xie, Zihao Tang, Xiaojin Lei, Chao Wang, Yujie Fu, Caiqiu Gao

**Affiliations:** ^1^ State Key Laboratory of Tree Genetics and Breeding Northeast Forestry University Harbin China; ^2^ Key Laboratory of Forest Plant Ecology Ministry of Education Northeast Forestry University Harbin China; ^3^ College of Chemistry Chemical Engineering and Resource Utilization Northeast Forestry University Harbin China

**Keywords:** *Betula platyphylla*, glycine‐rich RNA‐binding protein, *BpmiR396c*/*BpGRF3* regulatory module, gene regulatory, salt tolerance

## Abstract

Glycine‐rich RNA‐binding proteins (GRPs) have been implicated in the responses of plants to environmental stresses, but the function of *GRP* genes involved in salt stress and the underlying mechanism remain unclear. In this study, we identified *BpGRP1* (glycine‐rich RNA‐binding protein), a *Betula platyphylla* gene that is induced under salt stress. The physiological and molecular responses to salt tolerance were investigated in both BpGRP1‐overexpressing and suppressed conditions. *BpGRF3* (growth‐regulating factor 3) was identified as a regulatory factor upstream of *BpGRP1*. We demonstrated that overexpression of *BpGRF3* significantly increased the salt tolerance of birch, whereas the *grf3‐1* mutant exhibited the opposite effect. Further analysis revealed that BpGRF3 and its interaction partner, BpSHMT, function upstream of *BpGRP1*. We demonstrated that *BpmiR396c*, as an upstream regulator of *BpGRF3*, could negatively regulate salt tolerance in birch. Furthermore, we uncovered evidence showing that the BpmiR396c/BpGRF3 regulatory module functions in mediating the salt response by regulating the associated physiological pathways. Our results indicate that *BpmiR396c* regulates the expression of *BpGRF3*, which plays a role in salt tolerance by targeting *BpGRP1*.

## Introduction

Plant growth and productivity are under constant threat due to environmental challenges. Salt stress is a significant limiting factor that has a negative impact on various aspects of plant growth and development and on the quality and productivity of agroforestry (Safdar *et al*., 2019; Vishal *et al*., 2019; Yaldiz and Camlica, 2021). With the rapid development of a global society and economy and the acceleration of industrialization, poorly planned land utilization and development will further lead to continuous expansion of global saline and alkali land areas. Soil salinization makes it difficult to utilize a large portion of soil resources, which has become one of the serious factors affecting regional economic development and ecological restoration. It is therefore important to invest in the selection and development of salt‐tolerant plants.

Posttranscriptional modification is important for regulating the expression of defence‐related genes (Gough and Sadanandom, 2021). Posttranscriptional gene regulation involves several processes, including precursor mRNA (pre‐mRNA) splicing, nucleocytoplasmic transport of mRNA and mRNA stability and decay, and translation is mainly achieved either directly by ribonucleic acid (RNA)‐binding proteins (RBPs) or indirectly by RBPs modulating the function of other regulatory factors (Aguilar‐Garrido *et al*., 2022; Hajieghrari and Farrokhi, 2022; Shi and Grifone, 2021). Plants contain numerous RBPs, including glycine‐rich RNA‐binding proteins (GRPs) containing an RNA‐recognition motif (RRM) at the N‐terminus and a glycine‐rich region at the C‐terminus (Lu *et al*., 2019; Ma *et al*., 2021).

The first GRP was found in maize (*Zea mays*, Gomez *et al*., 1988), and since then, genes encoding homologous proteins have been consecutively isolated from a variety of plant species, such as *Arabidopsis thaliana* (Carpenter *et al*., 1994), *Medicago sativa* (Ferullo *et al*., 1997) and *Nicotiana tabacum* (Hirose *et al*., 1993). The expression of *GRPs* is regulated by a variety of external stimuli, including cold, drought stress and wounding stress (Ma *et al*., 2021; Shim *et al*., 2021; Xu *et al*., 2022). For instance, *GRPs* function as RNA chaperones during cold adaptation processes in rice and Arabidopsis plants. *OsGRP1* and *OsGRP4* rescue the growth defect of cold‐sensitive Arabidopsis *grp7* mutant plants under cold and freezing stress, whereas *OsGRP6* enhances the frost resistance of *grp7* (Kim *et al*., 2010). In addition, *AtGRP2*‐ or *AtGRP7*‐expressing transgenic rice plants show higher recovery rates and grain yields under drought stress than wild‐type plants (Yang *et al*., 2014). The overexpression of *OsGRP3* alleviates reactive oxygen species (ROS) accumulation by regulating ROS‐related gene mRNA stability under drought stress, which confers drought tolerance (Shim *et al*., 2021). However, less is known about the regulatory mechanisms of *GRPs* in defence responses (including salt stress) than about their physiological functions. Therefore, it is necessary to identify salt tolerance‐related *GRPs*.

Growth‐regulating factors (GRFs) are plant‐specific transcription factors that regulate stress‐related physiological and metabolic pathways by binding to the promoter sequences of multiple downstream stress‐related functional genes to activate or repress the expression of genes in response to stress (Omidbakhshfard *et al*., 2018). *AtGRF7* inhibits *AtDREB2A* expression by binding to the TGTCAGG element of the *AtDREB2A* promoter to maintain the balance between plant growth and resistance to drought and abscisic acid (ABA) stress, which enables plants to survive in an adversarial environment (Kim *et al*., 2012). *PdbGRF1* regulates the expression of *PdbPOD17* and *PdbAKT1* by binding to the DRE (‘A/GCCGAC’) in their respective promoters to increase ROS scavenging, reduce the extent of damage to the plasma membrane and ultimately enhance the salt stress response in *Populus davidiana* × *P. bolleana* (Liu *et al*., 2022a). In addition, GRF can regulate the expression of different target genes by interacting with different proteins and thus participate in different physiological and metabolic pathways. For example, in Arabidopsis, AtGRF5, in addition to forming a macromolecular complex with AtGIF1, positively regulates the leaf size by promoting and/or maintaining the cell proliferation activity of the leaf primordia (Horiguchi *et al*., 2005); this protein also interacts with DELLA to regulate leaf growth and root elongation and development through the gibberellic acid (GA) pathway and thus participates in the cold stimulus response (Ourania *et al*., 2020).

Various studies have indicated that the regulation of GRFs by miR396 plays an important role in plant growth and development and is involved in a variety of stress responses (Li *et al*., 2019; Pegler *et al*., 2021). In Arabidopsis, one of the *miR396* target genes, *AtGRF7*, functions as a repressor of stress‐responsive genes in the plant's abiotic stress response (Kim *et al*., 2012). *Sp‐miR396a‐5p* plays a positive regulatory role in the abiotic stress response by targeting the *NtGRF7*‐regulated expression of osmotic stress‐responsive genes (Chen *et al*., 2015). *Hpo‐miR396b* and *HpGRF6* may exert an antagonistic effect to negatively regulate the response of pitaya (*Selenicereus monacanthus*) to various abiotic stresses, such as low temperature, high temperature, NaCl and ABA (Li *et al*., 2019).

In this study, we cloned and functionally characterized a glycine‐rich RNA‐binding protein, BpGRP1, from *Betula platyphylla*, an important timber and landscaping tree species that has been widely used in plate processing, appliance manufacturing and plant medicine development (Guo *et al*., 2017). Our results showed that the overexpression of *BpGRP1* improved the tolerance of transgenic birch to salt stress. We further demonstrated that *BpGRF3*, an upstream regulator of *BpGRP1*, was regulated by *BpmiR396c* and was thus involved in the regulation of salt stress in birch. Based on the current data, we established a direct molecular link among *BpGRP1*, *BpGRF3* and *BpmiR396c* for improving the salt tolerance of birch.

## Results

### *BpGRP1* improves salt stress tolerance in transgenic birch

**Figure 1:**
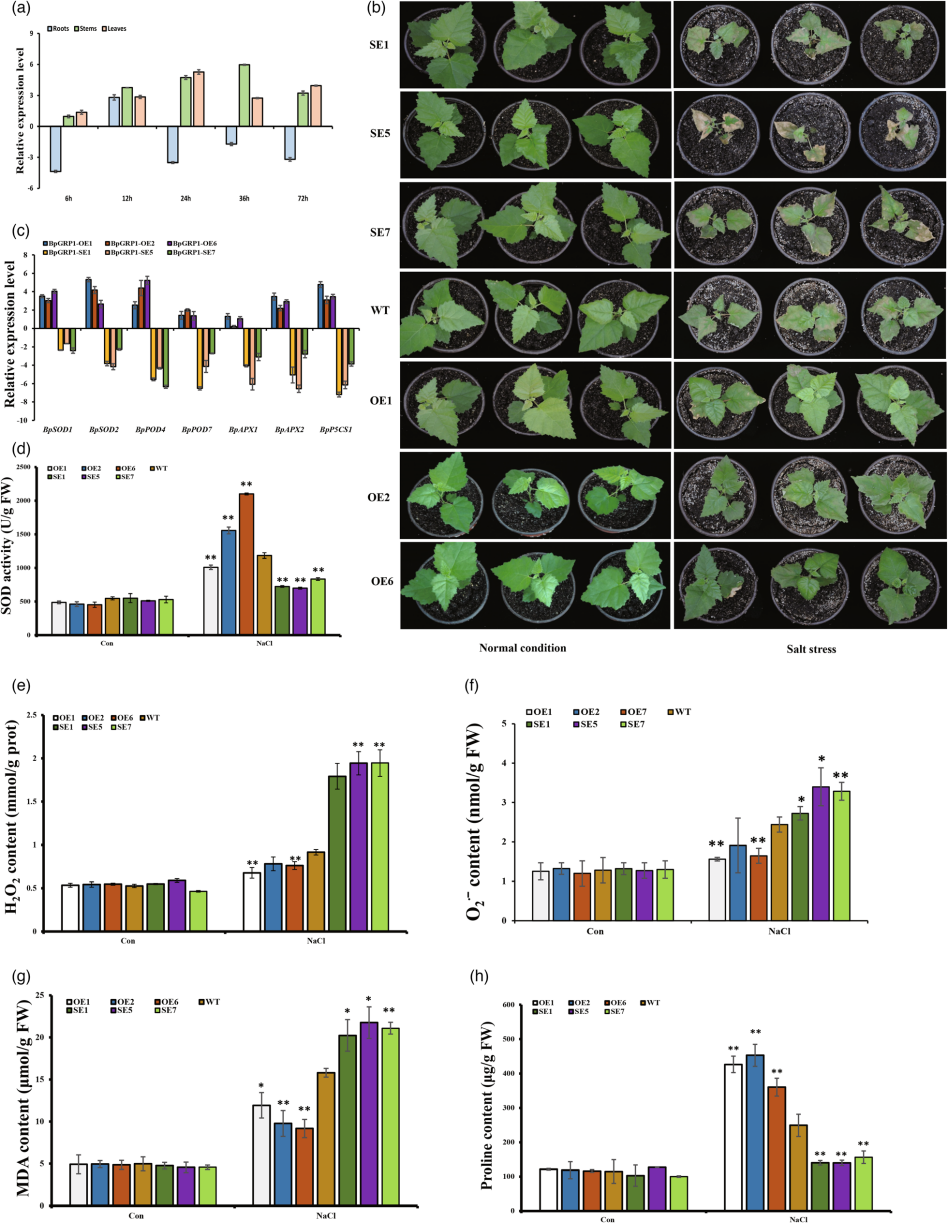
*BpGRP1* improves the salt stress tolerance of transgenic birch. (a) RT–qPCR analysis of *BpGRP1* expression during exposure to salt stress. (b) The growth of plants of the transgenic lines (OE lines, SE lines) and WT plants was compared under normal or salt stress conditions. Normal condition: normal growth conditions. Salt stress: treatment with 0.2 M NaCl for 14 d. (c) Analysis of the expression of salt‐related genes (*BpSODs*, *BpPODs*, *BpAPXs* and *BpP5CS1*) in WT, *BpGRP1*‐OE and *BpGRP1*‐SE plants. All expression values were log_2_‐transformed. (d–h) SOD activity, H_2_O_2_ content, O_2_^∙^ˉ content, MDA content and proline content. Control: plants grown under normal growth conditions. NaCl: treatment with 0.2 M NaCl for 7 d. The error bars indicate the standard deviations (SDs) of three biological replicates. * indicates *P* ≤ 0.05, and ** indicates *P* ≤ 0.01.

Due to its preferred growth conditions, birch usually exhibits distinctive and obvious symptoms under salt stress. To identify the critical genes that respond to salt stress, we analysed RNA‐Seq data under 200 mM NaCl treatment and identified many differentially expressed genes (Table S2), including the *BpGRP1* gene. RT–qPCR was employed to analyse the expression patterns of *BpGRP1* (GenBank accession number: BPChr06G07927) in different tissues at various time points under salt stress. The results showed that *BpGRP1* was significantly upregulated in stems and leaves after salt stress (Figure 1a).

To further determine the functional roles of *BpGRP1* in salt stress responses, *BpGRP1* transgenic lines were obtained (Figure S1). Soil‐grown transgenic birch plants, including OE, WT and SE lines, were exposed to salt to evaluate their stress responses. No substantial difference in phenotype was found among the OE, WT and SE lines under control conditions (Figure 1b), suggesting that *BpGRP1* does not affect the growth phenotype or growth rate of the plants. Under salt stress, the OE lines displayed significantly higher growth rates, had greener foliage and exhibited less wilting compared with WT plants. In contrast, the SE lines exhibited more severe leaf rolling and wilting (Figure 1b). Moreover, the expression of stress‐associated genes, including *BpSODs* (*BpSOD1*; *BpSOD2*), *BpPODs* (*BpPOD4*; *BpPOD7*), *BpAPXs* (*BpAPX1*; *BpAPX2*) and *BpP5CS1*, was significantly higher in the OE lines, whereas the expression levels of these genes were significantly lower in the SE lines (Figure 1c).

(i) The expression levels of *BpSODs*, *BpPODs*, *BpAPXs* and *BpP5CS1* were increased in the OE lines and decreased in the SE lines and (ii) the balance between ROS production and ROS removal is closely related to plant resistance to stress. Therefore, in this study, the activity of SOD was measured as scavenging capability, and the analysis showed that SOD activity was significantly higher in the OE plants than in the WT and SE plants following salt treatment but remained unaltered under normal conditions (Figure 1d). Moreover, the H_2_O_2_ and O_2_^∙^ˉ contents under unstressed conditions were similar among all the studied lines. Under salt stress, the trend of the changes in the H_2_O_2_ and O_2_^∙^ˉ contents was opposite to that found for SOD activity, i.e., the SE lines exhibited the highest H_2_O_2_ and O_2_^∙^ˉ contents, followed by the WT plants and then the OE lines (Figure 1e,f).

Malondialdehyde (MDA) is one of the end products of lipid peroxidation. To further analyse membrane lipid peroxidation in the studied lines, MDA levels were determined. Under normal conditions, the MDA content of the *BpGRP1* transgenic seedlings did not differ from that of the WT seedlings. However, after salt stress, the MDA content of the WT plants was significantly lower than that of the SE plants but significantly higher than that of the OE plants (Figure 1g). Furthermore, to investigate the role of *BpGRP1* in the salt stress response through the regulation of osmotic substances, the proline content was quantified and compared among the OE, WT and SE lines. Under normal conditions, no significant difference in the proline content was found among the studied plants, but after exposure to salt stress, the OE plants had the highest proline content, followed by the WT plants, and the SE plants had the lowest proline content (Figure 1h).

Taken together, these results demonstrated that *BpGRP1* positively regulates the salt stress response by affecting salt stress‐related gene expression, enhancing ROS scavenging capability, increasing proline levels and reducing MDA levels.

### BpGRF3 is a regulatory factor upstream of *BpGRP1*

**Figure 2:**
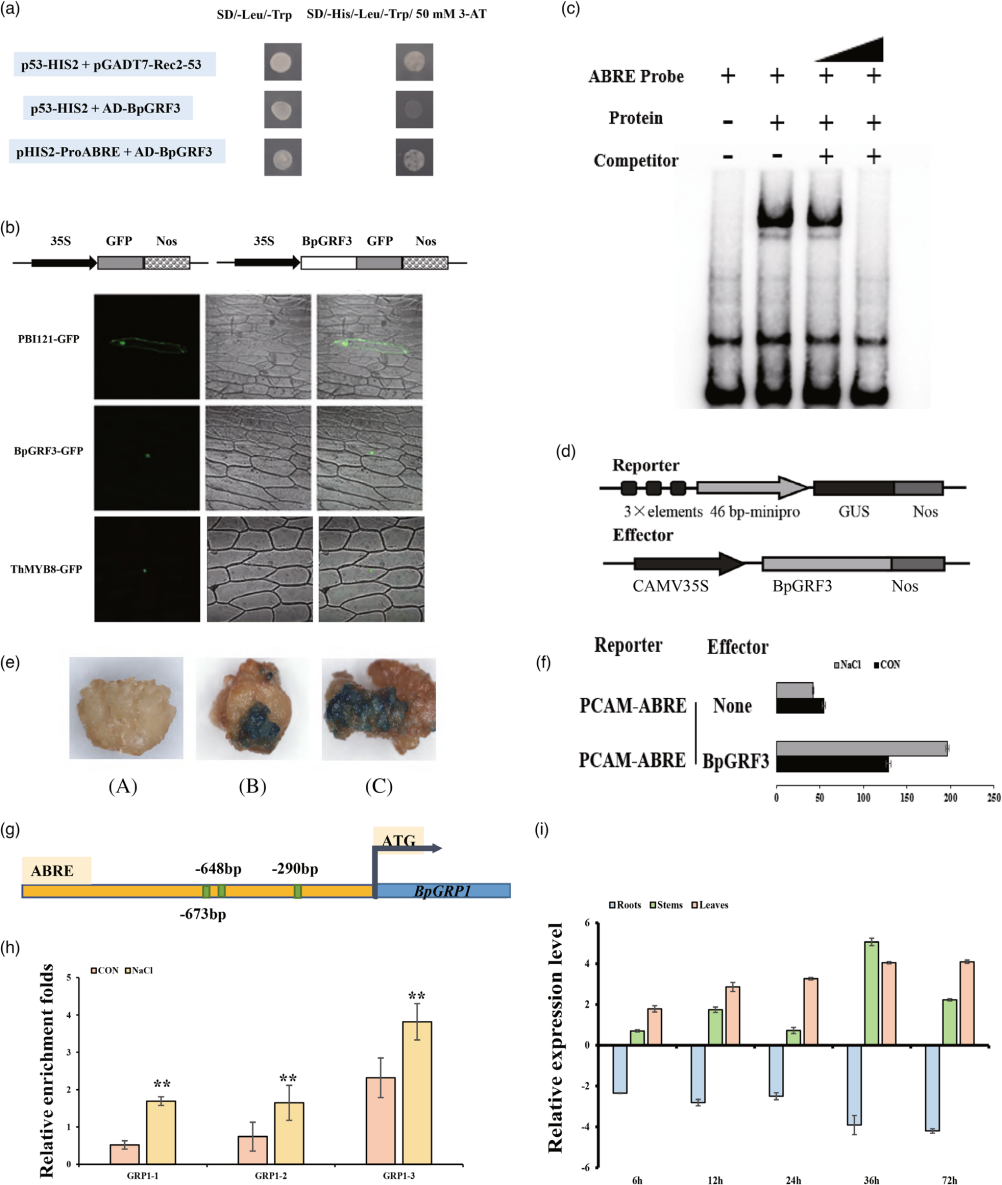
BpGRF3 regulates *BpGRP1* by directly binding to ABREs in vitro and in vivo. (a) A yeast one‐hybrid (Y1H) assay was used to verify the binding of BpGRF3 to ABREs. p53‐HIS2/pGADT7‐Rec2‐p53 was used as a positive control. pGADT7‐Rec‐BpGRF3/P53‐HIS2 was used as a negative control. (b) BpGRF3 was fused with the green fluorescence protein (*GFP*) gene under control of the 35S promoter, and 35S::GFP (control) was transiently expressed in onion epidermal cells via the particle bombardment method and visualized under a confocal microscope at 488 nm for the excitation of GFP and 507 nm longpass for emission. GFP, GFP fluorescence; Bright, bright field; Merge, merged bright field and fluorescence images. A nucleus‐localized MYB transcription factor, ThMYB8, was used as a positive control (Liu *et al*., 2020). (c) The binding of BpGRF3 to the ABRE in *BpGRP1* promoters was evaluated by EMSA. The biotin‐labelled probe was used as a negative control; the biotin‐labelled probe incubated with BpGRF3 protein was tested; and competitive probes were used at 10‐fold and 100‐fold (lack of biotin label). (d) Schematic diagram of the effector and reporter constructs used for coexpression in birch calli. (e) GUS staining of birch calli. (A) Transient expression of CAM‐ABRE alone; (B) transient expression of the cotransformed effector and reporter under normal conditions; (C) transient expression of the cotransformed effector and reporter under salt stress. (f) GUS activity assay showing the binding of the BpGRF3 protein to the ABRE in vivo. The values represent the means ± SDs of three biological replicates. (g) Distribution of the BpGRF3‐binding ABRE in the promoter of *BpGRP1*. (h) ChIP–qPCR analysis of the association of *BpGRF3* with *BpGRP1* promoters in vivo using an anti‐GFP tag antibody. The relative abundance of *BpGRF3*‐targeted promoter fragments in chromatin isolated under normal conditions or salt treatment conditions (0.2 M NaCl) for 24 h. The error bars indicate the standard deviations (SDs) of three biological replicates. * indicates *P* ≤ 0.05, and ** indicates *P* ≤ 0.01. (i) RT–qPCR analysis of *BpGRF3* expression under salt stress. The error bars indicate the standard deviations (SDs) of three biological replicates.

To investigate the role played by *BpGRP1* in the salt tolerance mechanism of birch, we analysed its promoter. A 2262‐bp sequence upstream of the *BpGRP1* translation start site was cloned from the birch genome sequence. A bioinformatics analysis of the *BpGRP1* promoter revealed numerous stress‐related consensus cis‐acting elements, including abiotic stress‐related elements and hormone stress‐related elements. The ABA‐responsive element (ABRE) with the highest frequency of 10 times (Figure S2) was selected as the target for identifying the possible upstream genes of *BpGRP1* by Y1H experiments. The results indicated that *BpGRF3* (growth‐regulating factor 3, GenBank accession number: BPChr01G18064) binds to the ABRE motif (Figure 2a).

We isolated the *BpGRF3* gene with two highly conserved structural domains (QLQ, Glu‐Leu‐Glu; WRC and Trp‐Arg‐Cys) at the N‐terminus in birch (Figure S3). Further subcellular localization analysis showed that BpGRF3‐GFP preferentially localized to the nucleus, whereas control GFP was distributed in both the cytoplasm and the nucleus (Figure 2b).

We further investigated whether *BpGRF3* was the upstream regulator of *BpGRP1*. An electrophoretic mobility shift assay (EMSA), transient expression analysis and chromatin immunoprecipitation (ChIP) were performed. We found reproducible binding of the *BpGRF3*‐maltose‐binding protein specifically to the ABRE motif (Figure 2c). The effector construct was transformed into calli of birch together with the ABRE motif reporter construct pCAM‐ABRE (Figure 2d). GUS activity determination showed that *BpGRF3* highly activated the expression of the reporter gene in the presence of the ABRE motif (Figure 2e,f). The ChIP–qPCR results were consistent with the EMSA results; that is, *BpGRF3* bound to the promoters of *BpGRP1* (Figure 2g,h). Furthermore, under salt stress, the expression pattern of *BpGRF3* showed an expression trend consistent with that found for *BpGRP1* (Figure 2i). Based on our experimental evidence, we confirmed that *BpGRP1* was also a direct target gene of *BpGRF3*.

### The overexpression of *BpGRF3* in birch improves tolerance to salt stress

**Figure 3:**
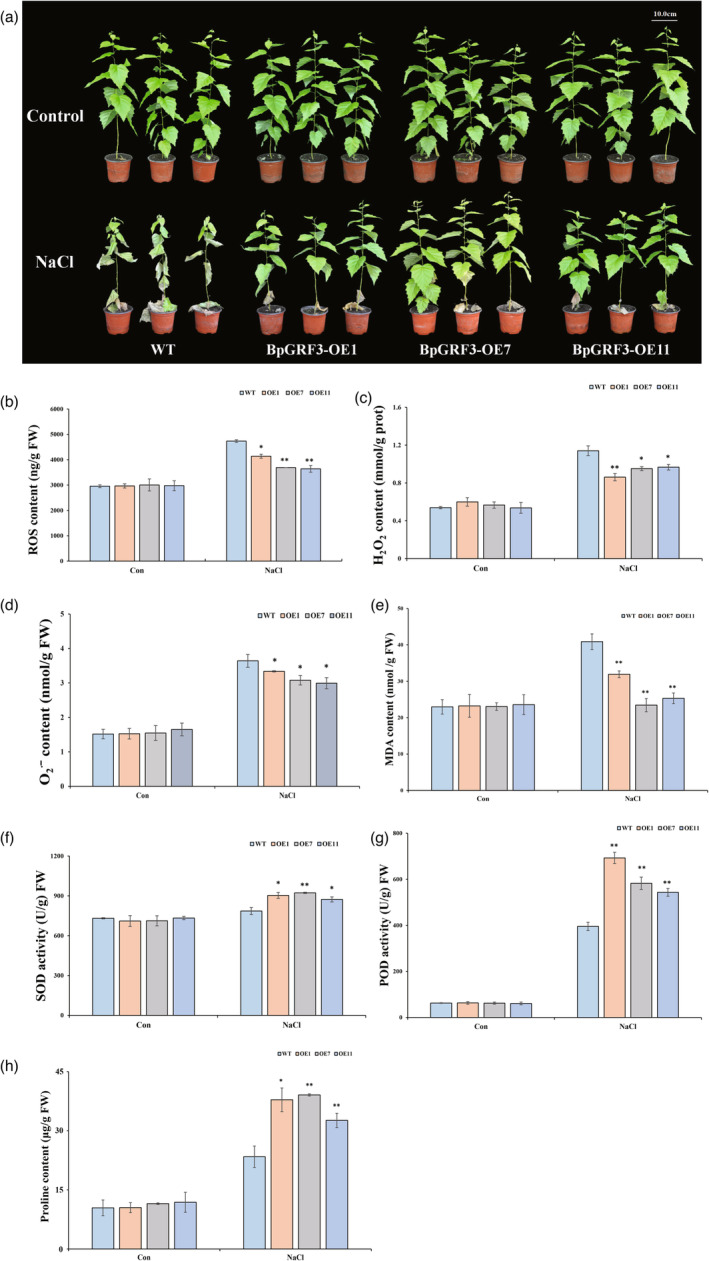
The overexpression of *BpGRF3* in birch improves tolerance to salt stress. (a) The growth of plants of the transgenic lines (OE lines) and WT plants was compared under normal or salt stress conditions. Control: plants grown under normal growth conditions. NaCl: treatment with 0.2 M NaCl for 14 d. (b–h) ROS content, H_2_O_2_ content, O_2_^∙^ˉ content, MDA content, SOD activity, POD activity and proline content. Control: plants grown under normal growth conditions. NaCl: treatment with 0.2 M NaCl for 7 days. The error bars indicate the standard deviations of three biological replicates. * indicates *P* ≤ 0.05, and ** indicates *P* ≤ 0.01.

To investigate the roles of *BpGRF3* in salt tolerance, we upregulated *BpGRF3* expression and assessed the success of the upregulation by RT–qPCR analysis. *BpGRF3* was significantly overexpressed in the OE lines (the expression levels in the OE1, OE7 and OE11 lines were 135.61, 23.70 and 79.71 times higher, respectively, than those in the WT line), indicating that the OE lines had been successfully established (Figure S4). We then compared the phenotypes of the *BpGRF3* (OE) lines with those of the WT cultured under both normal growth and salt stress conditions for 14 days. The *BpGRF3‐*OE lines displayed growth phenotypes similar to those of the *BpGRP1‐*OE lines. No significant difference in growth under normal growth conditions was found among the studied plants. However, under salt stress, the WT plants showed severely inhibited growth, with leaves that were withered, yellowed and curled and many leaves falling off the plants, whereas most of the OE lines showed normal growth with green and fully expanded leaves (Figure 3a).

To determine the salt tolerance mechanism mediated by *BpGRF3* at the physiological level, the ROS, H_2_O_2_, O_2_^∙^ˉ and MDA contents were measured. The trend for the changes in the ROS, H_2_O_2_, O_2_^∙^ˉ and MDA contents in the *BpGRF3‐*OE lines was consistent with that found in the *BpGRP1‐*OE lines. Under salt stress conditions, the levels of ROS, H_2_O_2_, O_2_^∙^ˉ and MDA were significantly higher in the WT plants than in the OE plants (Figure 3b–e). To investigate whether the reductions in ROS and lipid peroxidation were caused by altered antioxidant activity, the activities of superoxide dismutase (SOD) and peroxidase (POD) were studied. Under non‐saline conditions, no difference in SOD or POD activity was found between transgenic birch and WT plants. However, under salt stress, SOD and POD activities increased in all the studied lines. The activities of SOD and POD were significantly higher in the OE plants than in the WT plants (Figure 3f,g). In addition, under salt stress, the trend for the change in the proline content in the *BpGRF3‐*OE lines was similar to that found in the *BpGRP1‐*OE lines (Figure 3h).

**Figure 4:**
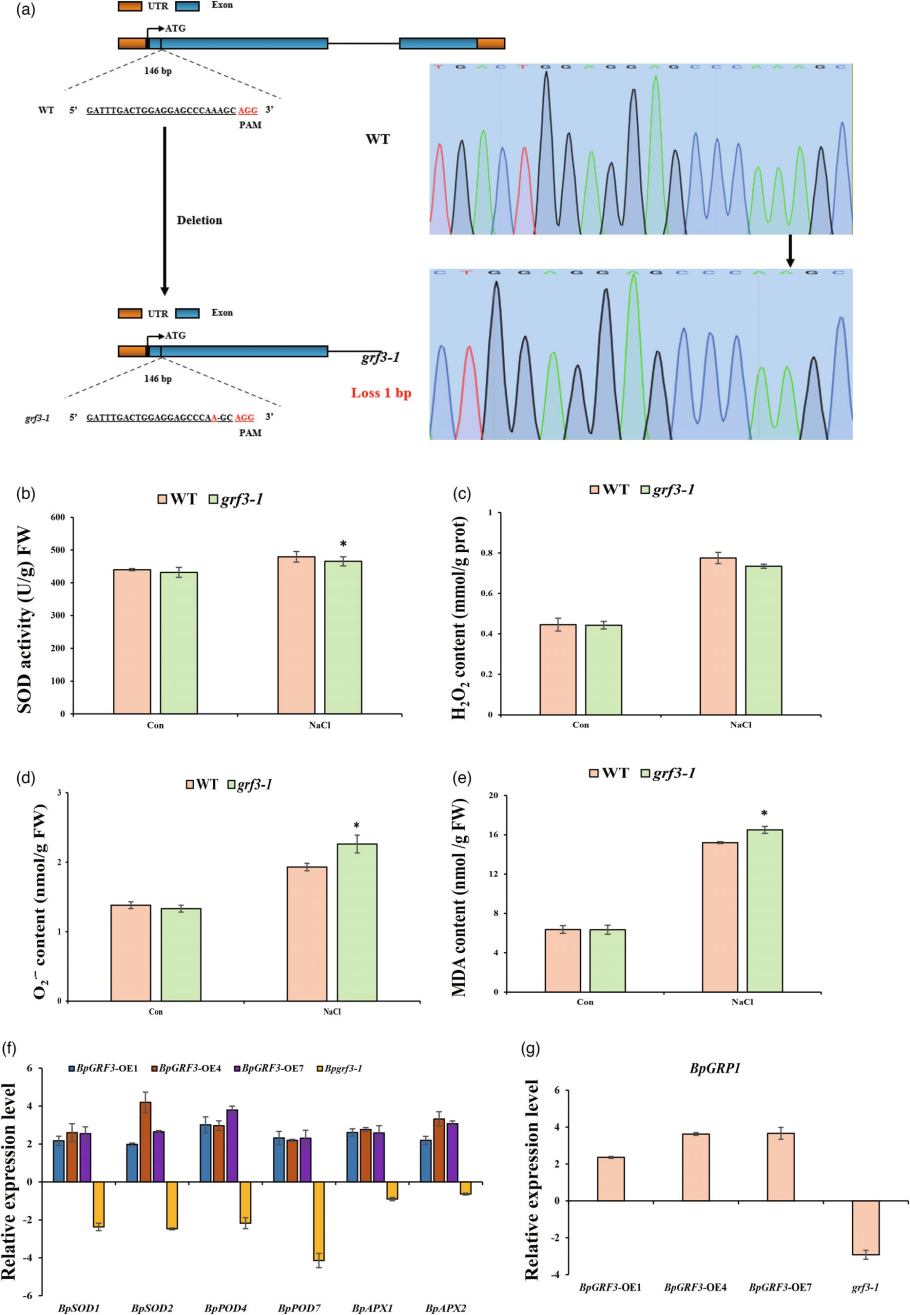
*BpGRF3* positively regulates salt tolerance in birch. (a) Gene structures of *BpGRF3* with a CRISPR/Cas9 target site designed in the exon. The black lines, orange strips and blue strips indicate introns, untranslated regions (UTRs) and exons, respectively. The nucleotide sequences indicate regions targeted by the gRNA designed in this study, and the nucleotides in red indicate proto‐spacer adjacent motifs (PAMs). (b–e) SOD activity, H_2_O_2_ content, O_2_^∙^ˉ content and MDA content. Con: plants grown under normal growth conditions. NaCl: treatment with 0.2 M NaCl for 2 d. The error bars indicate the standard deviations of three biological replicates. * indicates *P* ≤ 0.05, and ** indicates *P* ≤ 0.01. (f) Analysis of the expression of salt‐related genes (*BpSODs*, *BpPODs* and *BpAPXs*) in WT, *BpGRF3*‐OE and *grf3‐1* mutant plants. All expression values were log_2_‐transformed. (g) Analysis of the expression of *BpGRP1* in WT, *BpGRF3*‐OE and *grf3‐1* mutant plants. All expression values were log_2_‐transformed.

To further verify the function of *BpGRF3* in birch salt tolerance, a *BpGRF3*‐knockout mutant (*grf3‐1*) was generated using the CRISPR–Cas9 system (Figure 4a). Under salt stress conditions, the activity of SOD was significantly lower in the *grf3‐1* mutant than in the WT plants (Figure 4b). Accordingly, the trend of the changes in the O_2_^∙^ˉand MDA contents was the opposite of that found for SOD activity. Specifically, the *grf3‐1* mutant had higher O_2_^∙^ˉ and MDA contents than the WT (Figure 4d,e). Moreover, genes involved in the salt stress response, including *BpSOD1*, *BpSOD2*, *BpPOD4*, *BpPOD7*, *BpAPX1* and *BpAPX2*, were studied. Consistent with the physiological indicators related to stress resistance, the expression of *BpSODs*, *BpPODs* and *BpAPX*s was lowest in the *grf3‐1* mutant, moderate in the WT plants and highest in the *BpGRF3*‐OE lines (Figure 4f). Furthermore, the expression pattern of *BpGRP1* exhibited opposite trends in the *BpGRF3*‐OE lines and the *grf3‐1* mutant lines (Figure 4g).

Collectively, these findings suggest that *BpGRF3* enhanced salt stress tolerance in birch by regulating *BpGRP1* gene expression to activate stress‐associated physiological changes, such as enhancing the ROS scavenging capability, increasing the proline content and decreasing lipid peroxidation in cell membranes.

### BpGRF3 and BpSHMT coregulate the expression of *BpGRP1*

**Figure 5:**
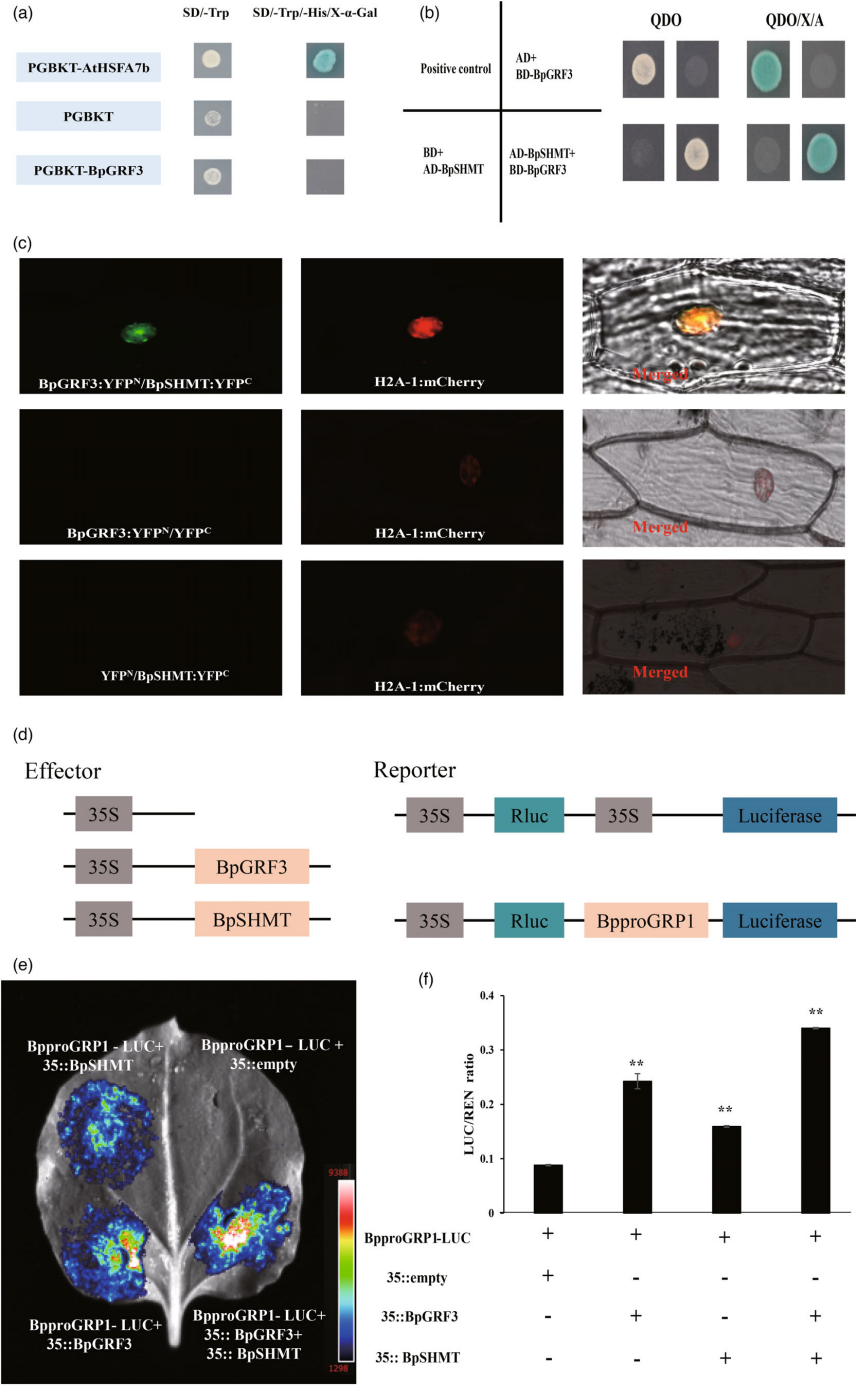
BpGRF3 interacts with BpSHMT. (a) Transcriptional activation activity of BpGRF3 in yeast. *BpGRF3* was cloned into the pGBKT7 vector to examine its role in gene activation. pGBKT7‐*AtHSFA7b* was used as a positive control (Zang *et al*., 2019). (b) A yeast two‐hybrid (Y2H) assay was used to identify the interaction of BpGRF3 with BpSHMT in yeast. QDO: SD/‐Leu/‐Trp‐His‐Ade, QDO/‐X/‐A: SD/‐Leu/‐Trp/‐His/‐Ade/X‐α‐Gal/AbA with 40 mg mL^−1^ X‐a‐Gal and 100 ng mL^−1^ AbA. pGBKT7‐53/pGADT7‐T was used as a positive control. (c) BiFC demonstrated that BpGRF3 interacted with BpSHMT in vivo. H2A‐1:m Cherry served as a nuclear marker. All experiments were performed with at least three independent biological replicates. (d) Schematic diagrams of effector and reporter constructs used for the dual luciferase assay. (e) Firefly luciferase complementation imaging assays of the interaction of BpGRF3 with BpSHMT in tobacco leaves. *A. tumefaciens* GV3101 strains harbouring BpproGRP1‐LUC and pROKII vectors were transfected into tobacco leaves. Luciferase imaging was performed 24 h after injection. (f) A dual‐luciferase reporter assay showed that BpGRF3 and BpSHMT positively regulated the expression of *BpGRP1*. Each value represents the mean ± SD of three biological replicates. * indicates *P* ≤ 0.05, and ** indicates *P* ≤ 0.01.

To investigate whether BpGRF3 functions as a transcriptional activator, full‐length *BpGRF3* was fused to the GAL4 DNA‐binding domain in the yeast expression vector pGBKT7. As shown in Figure 5a, little growth was observed in yeast transformed with the pGBKT7‐BpGRF3 constructs. These results indicated that BpGRF3 is a nuclear localization protein but has no transcriptional activation activity.

To determine how *BpGRF3* mediates *BpGRP1* and then participates in the regulation of salt tolerance, we conducted further investigation of the proteins that interacted with BpGRF3 by utilizing the Y2H system and proximity labelling (PL) approach in conjunction with mass spectrometry (MS)‐based quantitative proteomics. By screening the cDNA library of birch, a protein called BpSHMT (a serine hydroxymethyl transferase, GenBank accession number: BPChr12G11376) was identified as potentially interacting with BpGRF3. Additionally, the same BpSHMT protein was found through MS‐based quantitative proteomics (data not published; some data shown in Table S3). Based on the results obtained, it can be inferred that the BpSHMT protein, which responds to salt stress, has a high probability of interacting with BpGRF3 (Figure S5).

We tested this interaction by regenerating a full‐length BpSHMT‐pGADT7‐AD construct. Transformants harbouring BpGRF3‐pGBDT7‐BD and BpSHMT‐pGADT7‐AD exhibited a positive interaction on QDO/‐X/‐A solid medium, as indicated by a deep blue colour (Figure 5b). We then tested whether the BpGRF3–BpSHMT interaction occurs in planta by bimolecular fluorescence complementation (BiFC) in onion epidermal cells. The assays confirmed the interaction, as indicated by the detection of YFP signals from the interacting pairs, which colocalized with mCherry in the nucleus (Figure 5c).

We next explored the interaction of BpGRF3 and BpSHMT in the regulation of *BpGRP1*. The activation of *BpGRP1* promoter activity by both proteins was tested using an in vivo luciferase expression system. Both BpGRF3 and BpSHMT enhanced *BpGRP1* promoter activity, and stronger enhancement was obtained when the two proteins were expressed simultaneously (Figure 5d,e). In the LUC assay, coexpression of BpSHMT with BpproGRP1:LUC significantly increased LUC activity. The coexpression of BpGRF3 with the BpSHMT and BpproGRP1:LUC constructs further increased the LUC activity compared with that observed with only BpGRF3 or BpSHMT expression (Figure 5f).

Taken together, these results provide direct evidence showing that BpGRF3 and BpSHMT coregulate *BpGRP1* and that the presence of both BpGRF3 and BpSHMT enhances *BpGRP1* expression levels.

### The suppression of *BpGRF3* by *BpmiR396c* participates in the regulation of salt stress in birch

**Figure 6:**
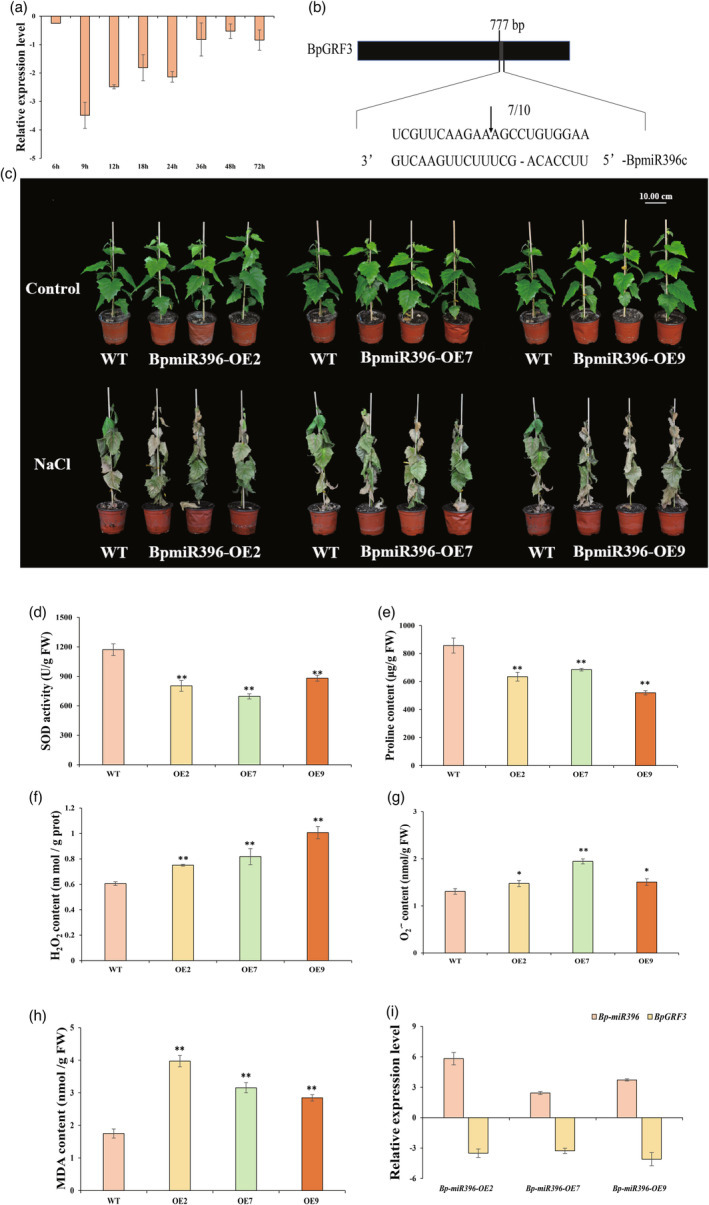
The overexpression of *BpmiR396c* inhibits the expression of *BpGRF3*, which enhances the salt tolerance sensitivity of transgenic birch plants. (a) RT–qPCR analysis of *BpmiR396c* expression under salt stress. All expression values were log_2_‐transformed. The error bars represent the standard deviations (SDs) of three biological replicates. (b) *BpmiR396c* cleavage sites in *BpGRF3*. The positions corresponding to the 5′ ends of the cleaved *BpGRF3* mRNAs determined by 5′ RACE and the frequency of 5′ RACE clones corresponding to each site are shown by arrows. (c) The growth of OE and WT plants under salt stress was compared. (d–h) SOD activity, proline content, H_2_O_2_ content, O_2_ˉ content and MDA content. NaCl: treatment with 0.2 M NaCl for 7 days. The error bars represent the standard deviations (SDs) of three biological replicates. * indicates *P* ≤ 0.05, and ** indicates *P* ≤ 0.01. (i) Relative expression of *BpmiR396c* and *BpGRF3* in plants of the WT and *BpmiR396c*‐OE lines. All expression values were log_2_‐transformed. The error bars represent the standard deviations (SDs) of three biological replicates.

Previous studies have shown that the target gene of *miR396* is *GRF*, which has been confirmed by RNA ligase‐mediated 5’‐rapid amplification of cDNA ends tests (5’ RLM‐RACE) (Peng *et al*., 2022). In this study, the *BpGRF3* transcript levels exhibited a pattern opposite to that found for *BpmiR396c* in response to salt (Figures 2i and 6a). To further validate the target site of *BpmiR396c*, we localized the *BpmiR396c*‐targeted cleavage sites in *BpGRF3* using 5’ RLM‐RACE. The results showed that the mRNAs of *BpGRF3* were cleaved by *BpmiR396c* between base pairs 11 and 12 (Figure 6b). Collectively, these findings support the notion that *BpmiR396c* directly cleaves *BpGRF3* transcripts to decrease their abundance.

To investigate the function of *BpmiR396c* in salt tolerance, the growth phenotype under salt stress was observed. No difference in the growth phenotype was found among the *BpmiR396c*‐OE lines (OE2, OE7 and OE9) and the WT plants under normal conditions. Under 0.2 M NaCl stress for 14 d, the leaves of the OE lines were withered, yellowed and curled, and many fell off the plants (Figure 6c). Moreover, the SOD activity and proline content were significantly lower in the OE plants than in the WT plants, whereas the H_2_O_2_, O_2_^∙^ˉ and MDA levels of the OE plants were significantly higher than those of the WT plants (Figure 6d–h).

To investigate the potential function of the BpmiR396c–BpGRF3 module, we isolated three independent transgenic lines (*BpmiR396c*‐OE2, 7, 9) with high expression levels (Figure S6): the *BpmiR396c* transcripts were increased by 56.49, 5.40 and 13.21 times in the OE2, OE7 and OE9 lines, respectively. However, the expression level of *BpGRF3* in the *Bp‐miR396*‐OE plants was significantly lower than that of the WT plants and was only 8.8%, 10.4% and 5.9%, respectively, of that in the WT plants (Figure 6i). Moreover, the phenotypes of the *BpmiR396c*‐OE and *BpGRF3*‐OE lines were opposite under salt stress. The *BpmiR396c*‐OE lines were more sensitive to salt stress, whereas the *BpGRF3*‐OE lines showed greater tolerance against these stresses (Figures 3a and 6c). Collectively, these results suggest that birch resistance to salt stress was negatively and positively modulated by *BpmiR396c* and *BpGRF3*, respectively.

## Discussion

### The stress‐inducible gene *BpGRP1* plays a positive role in salt stress tolerance

Marked changes in physiology, metabolism and, most notably, gene expression always occur when plants respond to adversity (Gupta *et al*., 2020; Wang *et al*., 2022a). Prior research has demonstrated that stress‐inducible genes are expressed at higher levels in plants accustomed to coping with salt stress (Li *et al*., 2022; Xing *et al*., 2020), and these genes include *GRP* genes (Tada *et al*., 2019). Furthermore, earlier studies have shown that the GRP protein exerts an influence on the growth and stress resilience of *Arabidopsis* plants under conditions of elevated salt levels and dehydration stress while also conferring tolerance to freezing (Kim *et al*., 2008). For example, *AtGRP2* promotes Arabidopsis seed germination and growth under salt and dehydration stress treatments (Su *et al*., 2009). *SvGRP1* enhances salt tolerance by increasing 3‐aminopropanoic acid, citramalic acid and isocitric acid levels (Tada *et al*., 2019).

In the present investigation, we successfully isolated glycine‐rich RNA‐binding protein 1 (BpGRP1) and subsequently identified it as a positive regulator in the salt stress response of birch plants (Figure 1a). Additionally, the modulation of *BpGRP1* expression in response to salt stress plays a crucial functional role in birch, as demonstrated in our investigation of transgenic birch plants. Notably, the phenotypic analysis of the *BpGRP1*‐OE and *BpGRP1*‐SE lines under salt stress conditions yielded distinct outcomes (Figure 1b), which was consistent with previous studies showing that genes with positive effects on salt resistance increase the salt tolerance of plants (Ma *et al*., 2021), indicating that *BpGRP1* positively regulates salt stress tolerance. Additional studies of the expression patterns of stress‐related genes in the *BpGRP1*‐OE and *BpGRP1*‐SE lines, including *BpSODs*, *BpPOD*s, *BpAPX*s and *BpP5CS1*, were performed. The findings revealed that *BpGRP1* had a positive impact on the salt stress response by influencing the expression of salt stress‐related genes (Figure 1c).

To elucidate the salt tolerance mechanism mediated by *BpGRP1* at the physiological level, we conducted an analysis of various physiological indicators. Our analysis of physiological indicators showed that the *BpGRP1*‐OE lines exhibited significantly improved SOD activity and proline contents and reduced levels of H_2_O_2_, O_2_^∙^ˉ and MDA, which enhanced their salt tolerance (Figure 1d–h). These results are in accordance with the capacity of *LbGST1* to confer salt tolerance in *Limonium bicolor* (Bunge) Kuntze through modulation of some physiological pathways (Wang *et al*., 2012). Interestingly, our findings regarding the analysis of physiological indicators of *BpGRP1*‐OE lines in response to salt stress contradict a previous report by Kim *et al*. (2007), who argued that (first) the H_2_O_2_ content is higher in transgenic plants than in wild‐type plants under salt stress and that (second) seed germination and seedling growth are hypersensitive to salt stress in *atRZ*‐*1a* (glycine‐rich RNA‐binding proteins) OE Arabidopsis.

The above‐described results suggest that homologous genes of *GRP* have different functions in different species and that their mechanisms of action are quite different, which is central to the significance of our investigation of the function of *BpGRP1* in birch.

### BpGRF3, as a regulatory factor upstream of *BpGRP1* , positively regulates salt stress in birch

Numerous studies have demonstrated the critical role of cis‐acting elements in regulating the expression of stress‐responsive genes under challenging environmental conditions, which enables plants to withstand and mitigate stress‐induced damage (Bo *et al*., 2022; Janiak *et al*., 2016; Wu *et al*., 2022). In this study, to investigate the role of *BpGRP1* in the salt tolerance mechanism of birch, its promoter was analysed. A bioinformatics analysis of the *BpGRP1* promoter revealed a significant number of stress‐related consensus cis‐acting elements, including those related to abiotic stress and hormone stress. Notably, the ABRE was found at the highest frequency, occurring 10 times (Figure S2). The results from a previous study revealed that ABRE is an essential binding site for the ABRE‐binding bZIP factor (*BnaABF2*) to activate *RD29B*, *RAB18* and *KIN2* transcription under drought and salt stress (Zhao *et al*., 2016). ANAC096 functions cooperatively with ABF2, and ABF4 regulates the expression of *RD29A* by targeting the ABRE cis‐acting element to confer both dehydration and osmotic tolerance (Xu *et al*., 2013).

Therefore, in this study, we focused on the ABRE and utilized it for cDNA library screening. Through Y1H experiments, the growth‐regulating factor BpGRF3 was identified (Figure 2a). EMSA and transient expression analysis were then performed to determine whether BpGRF3 could directly bind to ABREs in vitro/in vivo. BpGRF3 was able to bind to the ABRE, and the signal representing the complex was reduced or even disappeared in the presence of the competitor (Figure 2c). Similar binding was found by transient expression analysis, in which determination of GUS activity showed that *BpGRF3* greatly activated the expression of the reporter gene in the presence of the ABRE motif (Figure 2d–f). The ChIP–qPCR results were consistent with the EMSA and transient expression analysis results: *BpGRF3* bound to the promoters of *BpGRP1* (Figure 2g,h). Furthermore, under salt stress, the expression pattern of *BpGRF3* showed a consistent expression trend with that found for *BpGRP1* (Figure 2i). Collectively, these results suggest that *BpGRF3* is a regulatory factor upstream of *BpGRP1*.

A previous study showed that *PdbGRF1* acts as a key positive regulator of plant salt tolerance by regulating the expression of *PdbPOD17* and *PdbAKT1* (Liu *et al*., 2022a). To investigate the role of *BpGRF3* as a *BpGRP1* upstream regulatory factor in salt tolerance, the phenotype and physiological parameters of the BpGRF3‐OE line under salt stress were analysed. Similar to that found in the *BpGRP1*‐OE lines, overexpression of *BpGRF3* resulted in higher antioxidant enzyme activities and proline content and lower ROS and MDA levels under salt stress (Figure 3). However, the sensitivity of the *grf3‐1* mutant to salt stress may be attributed to its reduced SOD activity and elevated O_2_^∙^ˉ and MDA levels. Similarly, an analysis of the expression patterns of the stress‐related genes *BpSODs*, *BpPODs* and *BpAPXs* in *BpGRF3* transgenic plants was performed to explain the physiological differences between the BpGRF3‐OE lines and *grf3‐1* mutant lines. Furthermore, the expression pattern of *BpGRP1* exhibited opposite trends in the *BpGRF3*‐OE lines and *grf3‐1* mutant lines (Figure 4). Overall, the results show that *BpGRF3* enhances the salt tolerance of birch by combining with the ABRE of the promoter region to regulate the expression of the downstream gene *BpGRP1*.

Previous studies have revealed that *GRF* genes have the ability to recognize and bind specific elements, including ACTCGAC, CTTCTTC, CTGACA and TGTCAGG (Kim *et al*., 2012; Piya *et al*., 2020). In 84K poplar, PpnGRF5 enhances the expression of *PagCKX1p‐1* and *PagCKX1p‐2* by binding to the TGTCAG cis‐acting element, which results in enhancement of cell division and cell expansion in apical buds and young leaves and then enhancement of the growth and expansion of poplar leaves (Wu *et al*., 2021). The results from our previous study revealed that *PdbGRF1* enhances salt stress tolerance by regulating the expression of genes, including *PdbPOD17* and *PdbAKT1*, by binding to the dehydration‐responsive element (DRE) element (‘A/GCCGAC’) (Liu *et al*., 2022a). To date, few studies have investigated whether GRF‐regulating target genes participate in the abiotic stress response by targeting ABREs. Interestingly, the present study showed that BpGRF3 regulated *BpGRP1* to participate in salt stress regulation by directly binding to ABREs, which may be a previously unknown salt stress response pathway. Moreover, ABA has been demonstrated to mediate numerous physiological and adaptive responses resulting from adverse environmental conditions (Long *et al*., 2013). Our results showed that ABRE cis‐acting elements were highly enriched in the promoter region of *BpGRP1*. *BpGRP1* expression may be induced by ABA and may participate in salt stress regulation. Hence, elucidating the specific role of ABA in the regulation of birch salt tolerance is our next area of research interest and will be the focus of a study that will be conducted soon.

### BpGRF3 interacts with BpSHMT to positively regulate the expression of *BpGRP1*

Protein–protein interactions have been found to be useful for investigating complex biological activities and for understanding the ways in which external signals are perceived and transduced to trigger specific plant responses (Pazhamala *et al*., 2021; Struk *et al*., 2019). In Arabidopsis, AtGRF5 not only interacts with AN3 to regulate cell proliferation in leaves but also interacts with DELLA through the GA pathway to regulate leaf growth, root elongation and plant development and then participates in the response to cold stimuli (Horiguchi *et al*., 2005; Shahan, 2020). In this study, the fusion protein BpGRF3–pGBDT7‐BD did not activate the yeast reporter gene (Figure 5a). Whether *BpGRF3* mediates *BpGRP1* and then participates in the regulation of salt tolerance remains unclear. We speculated that BpGRF3 interacts with other proteins to form functional complexes and thus regulates the involvement of *BpGRP1* in salt stress. To verify this conjecture, the Y2H system and the PL approach combined with MS‐based quantitative proteomics were used to investigate the proteins interacting with BpGRF3. Fortunately, through the abovementioned two pathways, we identified a common potential BpGRF3‐interacting protein, BpSHMT. We then demonstrated the BpGRF3–BpSHMT interaction through a Y2H assay and BiFC in vitro/in vivo (Figure 5b,c). Previous studies have not found an interaction between GRF and SHMT. Thus, in this study, we were able to provide the first demonstration of the interaction between BpGRF3 and BpSHMT, and our subsequent resolution of the salt stress mechanism provides novel insight.

A previous study demonstrated that the *OsSHMT3* gene could be significantly induced by salt stress in rice and that overexpression of the *OsSHMT3* gene can significantly enhance the tolerance of Arabidopsis to salt stress (Mishra *et al*., 2019). The overexpression of *ApSHMT* in the freshwater cyanobacterium *Synechococcus elongatus* PCC7942 results in increased enzyme activities in serine biosynthetic pathways and enhanced salinity tolerance (Waditee‐Sirisattha *et al*., 2017). Similarly, in our study, *BpSHMT* exhibited the highest expression level under salt stress (Figure S5), implying that *BpSHMT* may play an important role in salt stress. Importantly, our results provide direct evidence showing that BpGRF3 and BpSHMT coregulated *BpGRP1* and that the presence of both BpGRF3 and BpSHMT enhanced *BpGRP1* expression (Figure 5d,f). BpSHMT is generally a mitochondrial protein (Jamai *et al*., 2009; Wu *et al*., 2015); however, in birch, BpGRF3 interacts with BpSHMT in the nucleus to regulate *BpGRP1* expression and then participates in the salt stress response. The pathway and mechanism by which BpSHMT enters the nucleus remain unclear and need further exploration.

### The overexpression of *BpmiR396c* inhibits the expression of *BpGRF3* , which is involved in the response of birch to salt stress

In plants, miR396 was previously recognized as a pivotal regulator governing various aspects of growth and development (Yu *et al*., 2020; Zhang *et al*., 2015). However, recent studies have also highlighted the significant role of miR396 in plant stress resistance (He *et al*., 2022; Pegler *et al*., 2021). For example, the *Sp‐miR396a‐5p* transcript levels are upregulated under salt and drought stresses (Chen *et al*., 2015); however, in *Sporobolus alterniflorus*, *miR396* is downregulated under salinity stress (Qin *et al*., 2014). It is thus clear that the highly conserved *miR396* has different functions in different species. The present study showed that *BpmiR396c* was downregulated in response to salinity stress (Figure 6a). Notably, the phenotypic and physiological parameters indicated that the expression of *BpmiR396c* can lead to increased sensitivity toward salt stress (Figure 6c–h), which suggests that *BpmiR396c* plays a negative role in regulating salt tolerance.

Prior research has indicated that the *GRF* gene is the target of *miR396*, a finding that has been corroborated through 5' RLM‐RACE analysis in various species, including *Oryza sativa* (Duan *et al*., 2016), *Panicum virgatum* (Liu *et al*., 2021b) and *Arabidopsis thaliana* (Beltramino *et al*., 2021). Similarly, in the present study, we sequenced a degradome library with 5' RLM‐RACE to confirm the cleavage sites. The *BpGRF3* transcripts were cleaved at the site complementary to *BpmiR396c* between base pairs 11 and 12 from the 5’ end of the miRNA (Figure 6b). Notably, the expression pattern of *BpGRF3* transcripts in response to salt stress was opposite to that of *BpmiR396c* (Figures 2i and 6a). The expression level of *BpGRF3* in the plants of the *Bp‐miR396*‐OE lines (OE2, OE7 and OE9) was significantly lower than that in the WT plants and was only 8.8%, 10.4% and 5.9%, respectively, of that in the WT plants (Figure 6h). Furthermore, in contrast to the suppressive effect of *msi‐miR164g* on drought stress, the overexpression of *MsNAC022* has been shown to enhance drought tolerance in transgenic Arabidopsis and apple (Peng *et al*., 2022). Similarly, we observed contrasting phenotypes between the *BpmiR396c*‐OE lines and the *BpGRF3*‐OE lines under salt stress (Figures 3a and 6c). Collectively, these results suggest that the resistance of birch to salt stress is negatively and positively modulated by *BpmiR396c* and *BpGRF3*, respectively.

**Figure 7:**
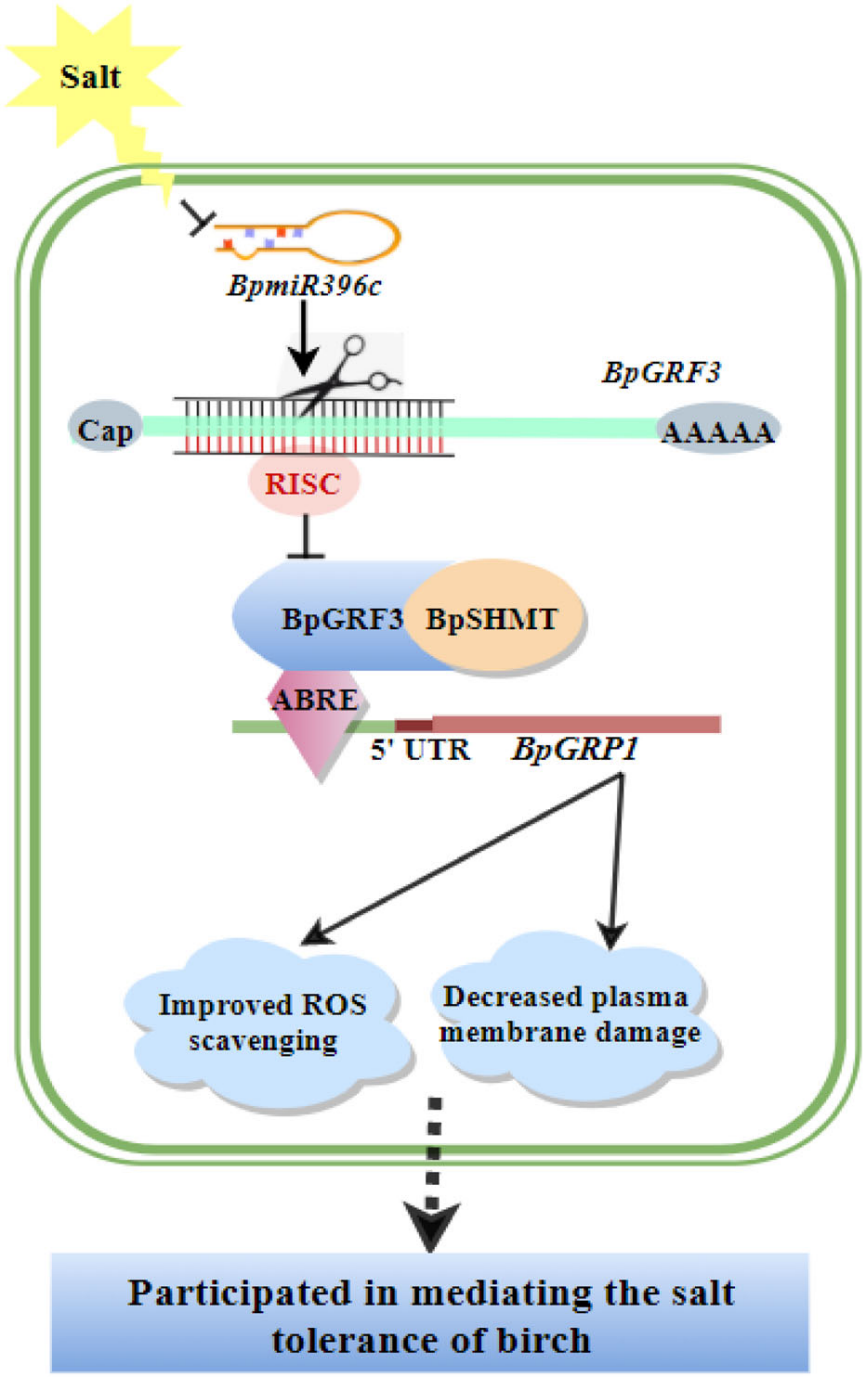
A putative model for the mediation of the transcriptional regulation of *BpGRP1* by the *BpmiR396c*‐*BpGRF3* module. This model may provide a new perspective for understanding the mechanism of birch salt tolerance. Under salt stress, a decrease in *BpmiR396c* diminishes cleavage of the downstream *BpGRF3* transcripts, and the encoded protein activates transcription of the downstream gene *BpGRP1*. With the interaction between BpGRF3 and BpSHMT, *BpGRP1* is activated to regulate the associated physiological pathways, which increases the ROS scavenging ability, reduces the degree of damage to the plasma membrane and ultimately enhances the salt stress response in birch.

Based on the above‐described findings, we propose a putative model of salt tolerance in birch mediated by the *BpmiR396c*–*BpGRF3*–*BpGRP1* module, which may provide a new perspective for understanding the mechanism of birch salt tolerance (Figure 7). Under salt stress, a rapid decrease in *BpmiR396c* levels alleviated the cleavage of *BpGRF3* transcripts, and the encoded transcription factor interacts with BpSHMT to form functional complexes that in turn activate *BpGRP1* transcription. Through the interaction between BpGRF3 and BpSHMT, *BpGRP1* is activated to regulate the associated physiological pathways, which increases the ROS scavenging ability, reduces the degree of damage to the plasma membrane and ultimately enhances the salt stress response in birch.

## Materials and Methods

### Plant materials and growth conditions

Birch seedlings (transgenic plants and wild‐type plants were used in this study) were grown in a greenhouse under a 16 h light/8 h dark photoperiod under 400 μmol photons m^−2^ s^−1^ irradiation at 25 °C (Tan *et al*., 2020). Three‐month‐old plants were watered with a solution of 0.2 M NaCl, and tissues were collected at 6, 12, 24, 36 and 72 h post‐watering. Seedlings watered with fresh water were harvested at the corresponding time points as controls.

### RNA extraction, cDNA synthesis and RT–qPCR analysis

RNA extraction, cDNA synthesis and RT–qPCR were performed as previously described (Liu *et al*., 2018) with a few modifications. Briefly, total RNA was extracted using the Plant RNeasy Extraction Kit (BioTeKe, Beijing, China, Cat. No. RP3302). First‐strand cDNA synthesis was conducted using *EasyScript*® First‐Strand cDNA Synthesis SuperMix (TransGen Biotech, Beijing, China, Cat. No. AE301‐02). Real‐time RT–PCR (RT–qPCR) was performed using TransStart® Top Green qPCR SuperMix (TransGen Biotech, Beijing, China, Cat. No. AQ131‐04). A qTOWER 3G Cycler and qPCR software (Analytik Jena, Germany) were used to perform the experiment and analyse the data. The 2^−ΔΔCt^ method was used to determine the relative abundance of transcripts (Livak and Schmittgen 2001). Ubiquitin (UBQ, FG065618) and *tubulin* (TU, FG067376) were used as internal controls (Lei *et al*., 2020; Liu *et al*., 2018).

### Plasmid construction and birch transformation

*BpGRP1*, *BpGRF3* and *Bp‐miR396c* were amplified by PCR with KOD DNA polymerase (TOYOBO, Osaka, Japan), gene‐specific primers and birch cDNAs. The full‐length coding sequences of *BpGRP1* and *BpGRF3* were cloned into the pROKII vector under control of the cauliflower mosaic virus 35S promoter (35S) for overexpression. The *Bp*‐miR396c precursor was introduced into the PCAMBIA1302 vector under control of the 35S CaMV promoter to overexpress *Bp*‐miR396c (Sahito *et al*., 2017). In addition, a truncated inverted‐repeat cDNA of *BpGRP1* (200 bp) was ligated into pFGC5941, an RNAi vector flanking the intron of *Chsa* (encoding chalcone synthase A), which yielded the pFGC5941::*BpGRP1* construct to silence the expression of *BpGRP1*.

Knockout mutants of *BpGRF3* were generated using the CRISPR–Cas9 system (Liu *et al*., 2022b). The sgRNA sequences were selected using CRISPR‐P2.0 (http://crispr.hzau.edu.cn/cgi‐bin/CRISPR2/CRISPR). The sgRNA sequences were synthesized and inserted into the pEgP237‐2A‐GFP vector.

All plasmids were introduced into *Agrobacterium tumefaciens* strain EHA105 for birch transformation as described previously (Guo *et al*., 2017). All transgenic lines were verified by PCR and RT–qPCR analysis. The primers are listed in Table S1.

### Assessment of the salt tolerance of transgenic birch

NaCl treatment was conducted to mimic salt stress conditions and to elucidate the function of *BpGRP1*, *BpGRF3* and *Bp‐miR396c* in the salt‐tolerance response of birch. To observe the growth of birch plants, plants of similar size (height of approximately 5 cm) from the wild‐type (WT) and all transgenic birch lines (*BpGRP1*, *BpGRF3* and *Bp‐miR396c* lines) were transplanted into 6‐inch pots containing a perlite/vermiculite/soil mixture at a ratio of 1:1:4 (*v*/*v*) and were grown in a greenhouse. After two months, the roots of the plants were watered with 0.2 M NaCl, and plants watered with well water served as controls. After treatment for 14 d, the plant phenotype was observed and photographed.

The plants of WT and transgenic (*BpGRP1*, *BpGRF3* and *Bp‐miR396c*) birch lines that had been grown in soil for two months were watered with a solution of 0.2 M NaCl for 7 d. Well‐watered plants were used as controls. The leaves of different lines were collected for the determination of physiological indices. The ROS, SOD, POD, MDA, H_2_O_2_, O_2_^∙^ˉ and proline levels were determined using a biological engineering kit from Nanjing Jiancheng Institute.

### Subcellular localization analysis of transiently expressed fusion proteins

The coding sequence of *BpGRF3* was fused in frame to the amino terminus of the enhanced green fluorescent protein (eGFP) coding sequence under control of the CaMV35S promoter, as previously described (Liu *et al*., 2020, 2022a). As a control, GFP transcribed using the CaMV 35S promoter (35S::GFP) was used. Particle bombardment (Bio‐Rad, Hercules, CA, USA) was used to introduce the constructs into onion epidermal cells. Confocal laser‐scanning microscopy (LSM700, Zeiss, Jena, Germany) was then used to analyse the transformed cells. The primers are listed in Table S1.

### Protein interaction

The full‐length coding region of *BpGRF3* was fused into the pGBDT7‐BD vector, and yeast two‐hybrid (Y2H) assays were performed as previously described (Liu *et al*., 2022b; Wang *et al*., 2022b). In brief, the *BpGRF3–*pGBDT7‐BD recombinant plasmids were transformed into Y2HGold yeast competent cells, and the transformed yeast competent cells were then placed on synthetic dropout SD/‐Trp and SD/‐Trp/‐His with 40 mg mL^−1^ X‐α‐Gal media to detect their growth status at 30 °C. The specific procedure used for PL combined with MS‐based quantitative proteomics was described by Yang *et al*. (2021).

Full‐length *BpSHMT* (serine hydroxymethyl transferase) was fused to the GAL4‐activating domain (AD) in the pGADT7 vector. *BpGRF3‐*pGBDT7‐BD and *BpSHMT‐*pGADT7‐AD were cotransformed into Y2HGold yeast competent cells. The transformed yeast cells were spread on SD/‐Leu/‐Trp‐His‐Ade and SD/‐Leu/‐Trp/‐His/‐Ade/X‐α‐Gal/AbA with 40 mg mL^−1^ X‐a‐Gal and 100 ng mL^−1^ aureobasidin A (AbA).

For BiFC assays, the *BpGRF3* and *BpSHMT* coding regions were infused into the pENTER/D‐TOPO vector and then recombined with nEYFP/pUGW2 and cEYFP/pUGW2, respectively (Wang *et al*., 2022b). *BpGRF3*:YFP^N^/*BpSHMT*:YFP^C^, *BpGRF3*:YFP^N^/YFP^C^ and YFP^N^/*BpSHMT*:YFP^C^ were cotransformed with H2A‐1:mCherry into onion epidermal cells. After 24 h of coculture, the fluorescence signals were observed using a laser‐focusing microscope (Zeiss LSM700). The primers are listed in Table S1.

### Yeast one‐hybrid (Y1H) assay

The coding sequence of *BpGRF3* was cloned into the pGADT7‐Rec2 vector (Clontech) to generate *BpGRF3*–pGADT7‐Rec2 constructs. The ABRE in the *BpGRP1* promoter was inserted into the pAbAi vector (Clontech) immediately upstream of the *AUR1‐C* gene. These constructs were cotransformed into the *Saccharomyces cerevisiae* strain Y187, and the Y1H assay was conducted as previously described (Liu *et al*., 2020). The primers are listed in Table S1.

### EMSA

The full‐length coding sequence of *BpGRF3* was cloned into the pMAL‐c5X vector and transformed into *Escherichia coli* strain ER2523 (NEB Express, Ipswich, MA, USA). An oligonucleotide probe containing the ABRE (ACGTG) motif derived from the *BpGRP1* promoter was labelled with biotin at its 5’ end by using an electrophoretic mobility shift assay (EMSA) Probe Biotin Labeling Kit (Beyotime, China). EMSAs were carried out with a chemiluminescence‐based EMSA kit (Beyotime, China). The primers are listed in Table S1.

### Detection of beta‐glucuronidase (GUS) activity

For the construction of reporter vectors, the ABRE motif sequence with three tandem copies was fused with a 46‐bp minimal 35S promoter to drive a *GUS* reporter gene. The effector, namely, the *BpGRF3* overexpression vector (35S::*BpGRF3*), was transformed into birch calli together with each reporter. To normalize the transformation efficiency, the 35S::Luc vector was also cotransformed. GUS activity was assessed as described by Jefferson (1987). For each GUS activity assay, three biological replicates were analysed, and three technical replicates were performed for each biological replicate. The primer sequences used for vector construction are shown in Table S1.

### ChIP‐qPCR analysis

The 35S::*BpGRF3*‐GFP construct was transiently transformed into birch for the ChIP assay, which was performed as described previously, with some modifications (Zhang *et al*., 2016; Zhao *et al*., 2020). Briefly, proteins and DNA were cross‐linked using formaldehyde (3% *v*/*v*) for 5 min. The purified DNA–protein crosslinks (DPCs) were sonicated and immunoprecipitated with an anti‐GFP antibody. The purified DPCs were also immunoprecipitated with an anti‐human influenza haemagglutinin (HA) antibody, which served as the negative control. Chromatin before immunoprecipitation was used as the input control. ChIP–qPCR was performed to study the enrichment of the target DNA sequence. The fold enrichment of ChIP–qPCR was calculated according to the method described by Haring *et al*. (2007). For each transfection, three biological replicates were conducted, and three technical replicates were conducted for each biological replicate. The ChIP–qPCR primers are shown in Table S1.

### Dual‐luciferase assay

The full‐length coding regions of *BpGRF3* and *BpSHMT* were fused into the pROKII vector, and the promoter sequence of *BpproGRP1* was fused into the pGreen 0800‐LUC vector (Wang *et al*., 2022b). 35S::*BpGRF3*/35S::*BpSHMT*/*BpproGRP1*‐0800LUC, 35S::*BpGRF3*/*BpproGRP1*‐0800LUC and 35S::*BpSHMT*/*BpproGRP1*‐0800LUC were coinfiltrated into the leaves of 6‐week‐old *Nicotiana benthamiana*. 35S::empty/*BpGRP1*‐0800LUC was used as a negative control. Luciferase activity was assayed using the Dual‐Luciferase® Reporter Assay System (Promega, Madison, USA, Cat. No. E1910) and then subjected to chemiluminescence using a fully automatic imaging analysis system (Tanon 5200). The primers used for the LUC assays are listed in Table S1.

### Validation of cleavage sites of *BpGRF3*

The 5' RLM‐RACE of the birch total RNA was performed using the GeneRacer kit (Invitrogen, Carlsbad, CA, USA), as described previously (Liu *et al*., 2021a). The amplification products were gel‐purified and cloned into the pCloneEZ vector (CloneSmarter). Ten independent clones were picked for sequencing.

### Statistical analysis

The data were analysed by one‐way ANOVA with Statistical Product and Service Solutions (SPSS) 16.0 (IBM Corp., Armonk, NY, USA) to determine significance. Statistical significance was defined as follows: *P ≤ 0.05 and **P ≤ 0.01.

## Author contributions

Cai‐Qiu Gao, Yu‐Jie Fu and Zhong‐Yuan Liu designed the research. Zhong‐Yuan Liu, Teng‐Qian Zhang, Rui‐Ting Xu, Ya‐Ting Han, Zi‐Hao Tang and Wen‐Fang Dong conducted the experiments. Qing‐Jun Xie, Chao Wang, Xiao‐Jin Lei, Bai‐Chao Liu and Zhong‐Yuan Liu analysed the data. Cai‐Qiu Gao, Yu‐Jie Fu and Zhong‐Yuan Liu wrote the manuscript with input from all the coauthors.

## Conflict of interest

The authors declare no conflicts of interest.

## Supporting information


**Figure S1.** Expression of *BpGRP1* in transgenic birch plants. (a) RT–qPCR analysis of the relative expression of *BpGRP1* in the OE lines. (b) RT–qPCR analysis of the relative expression of *BpGRP1* in the SE lines. OE lines: lines overexpressing *BpGRP1*. SE lines: *BpGRP1*‐silenced lines. All expression values were log_2_‐transformed. The error bars represent the standard deviations (SDs) of three biological replicates.
**Figure S2.** Cis‐acting elements in the *BpGRP1* promoter. (a) Locations of cis‐acting elements in the *BpGRP1* promoter. (b) Description and statistics of cis‐acting elements.
**Figure S3.** Sequence and phylogenetic analysis of the BpGRF3 protein. (a) Multiple sequence alignment of BpGRF3 and GRF proteins from other species. The black line indicates the QLQ domain and the WRC domain. (b) Phylogenetic analysis of BpGRF3 and GRF proteins from different plant species. The full names and accession numbers of the proteins analysed are listed in Table S4.
**Figure S4.** Expression of *BpGRF3* in WT and transgenic birch. RT–qPCR analysis of the relative expression of *BpGRF3* in the *BpGRF3‐*OE lines. All expression values were log_2_‐transformed. The error bars represent the‐standard deviations (SDs) of three biological replicates.
**Figure S5.** Analysis of *BpSHMT* gene expression under salt stress. All expression values were log_2_‐transformed. The error bars represent the standard deviations (SDs) of three biological replicates.
**Figure S6.** Expression of *BpmiR396c* in WT and *BpmiR396c*‐OE lines. All expression values were log_2_‐transformed. The error bars represent the standard deviations (SDs) of three biological replicates.

**Table S1.** List of primers used in this study.
**Table S2.** Differentially expressed genes in birch under salt stress.
**Table S3.** MS‐based quantitative proteomics data for the identification of GRF3‐interacting proteins.
**Table S4.** Full names and accession numbers of the proteins used for sequence and phylogenetic analyses.

## Data Availability

The data that support the findings of this study are available from the corresponding author upon reasonable request.
