# Supplementary material for: BpGRP1 acts downstream of BpmiR396c/BpGRF3 to confer salt tolerance in Betula platyphylla
Source: Plant Biotechnol J. 2023 Sep 13;22(1):131–47. doi: 10.1111/pbi.14173 (PMC10754015; doi:10.1111/pbi.14173)
Supplement: Supplementary file 1 — Figure S1. Expression of BpGRP1 in transgenic birch plants. (a) RT–qPCR analysis of the relative expression of BpGRP1 in the OE lines. (b) RT–qPCR analysis of the relative expression of BpGRP1 in the SE lines. OE lines: lines overexpressing BpGRP1. SE lines: BpGRP1‐silenced lines. All expression values were log2‐transformed. The error bars represent the standard deviations (SDs) of three biological replicates. Figure S2. Cis‐acting elements in the BpGRP1 promoter. (a) Locations of cis‐acting elements in the BpGRP1 promoter. (b) Description and statistics of cis‐acting elements. Figure S3. Sequence and phylogenetic analysis of the BpGRF3 protein. (a) Multiple sequence alignment of BpGRF3 and GRF proteins from other species. The black line indicates the QLQ domain and the WRC domain. (b) Phylogenetic analysis of BpGRF3 and GRF proteins from different plant species. The full names and accession numbers of the proteins analysed are listed in Table S4. Figure S4. Expression of BpGRF3 in WT and transgenic birch. RT–qPCR analysis of the relative expression of BpGRF3 in the BpGRF3‐OE lines. All expression values were log2‐transformed. The error bars represent the‐standard deviations (SDs) of three biological replicates. Figure S5. Analysis of BpSHMT gene expression under salt stress. All expression values were log2‐transformed. The error bars represent the standard deviations (SDs) of three biological replicates. Figure S6. Expression of BpmiR396c in WT and BpmiR396c‐OE lines. All expression values were log2‐transformed. The error bars represent the standard deviations (SDs) of three biological replicates. [file PBI-22-131-s001.pdf]

BpGRP1* acts downstream of *BpmiR396c/BpGRF3* to confer salt tolerance in *Betula platyphylla

Zhongyuan Liu[a,b,c], Tengqian Zhang[a], Ruiting Xu[a], Baichao Liu[a], Yating Han[a], Wenfang Dong[a], Qingjun Xie[a], Zihao Tang[a], Xiaojin Lei[a], Chao Wang[a], Yujie Fu[b,c,*], Caiqiu Gao[a*]

Supporting information

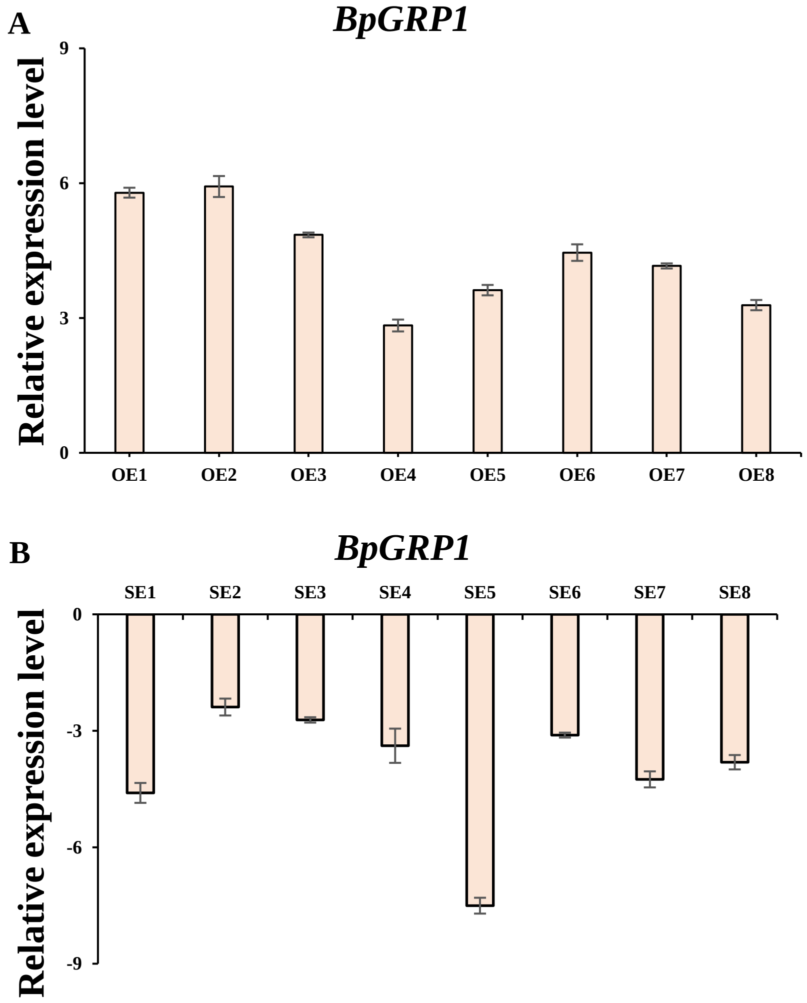

Supplemental Figure S1

Expression of *BpGRP1* in transgenic birch plants. (A) RT–qPCR analysis of the relative expression of *BpGRP1* in the OE lines. (B) RT–qPCR analysis of the relative expression of *BpGRP1* in the SE lines. OE lines: lines overexpressing *BpGRP1*. SE lines: lines with *BpGRP1* silencing. All expression values were log2 transformed. The error bars represent the standard deviations (SD) of three biological replicates.

A

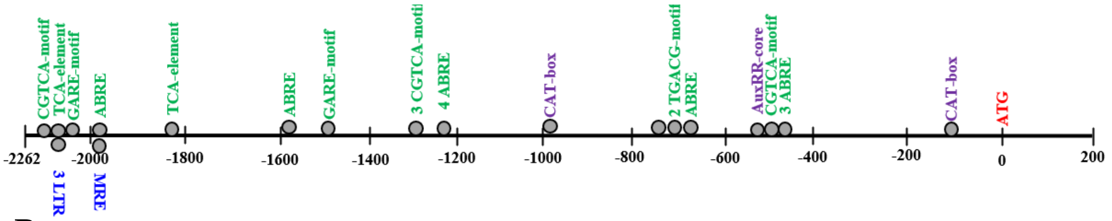

B

Class	Element	Description	Number
Hormone	ABRE	cis-acting element involved in the abscisic acid responsiveness	10
	CGTCA-motif	cis-acting regulatory element involved in the MeJA-responsiveness	7
	TCA-element	cis-acting element involved in salicylic acid responsiveness	2
	GARE-motif	gibberellin-responsive element	2
Stress	LTR	cis-acting element involved in low-temperature responsiveness	3
	MRE	MYB binding site involved in light responsiveness	1
Development	AuxRR-core	cis-acting regulatory element involved in auxin responsiveness	1
	CAT-box	cis-acting regulatory element related to meristem expression	2

Supplemental Figure S2

Cis-acting elements in the *BpGRP1* promoter. (A) The locations of cis-acting elements in the *BpGRP1* promoter. (B) Description and statistics of cis-acting elements.

A

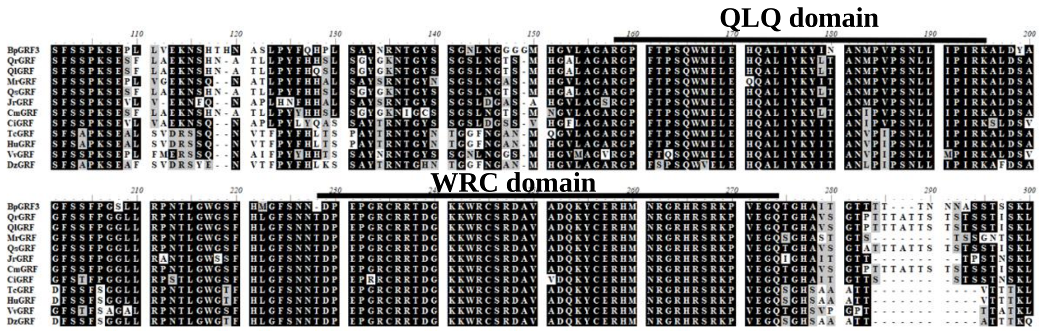

B

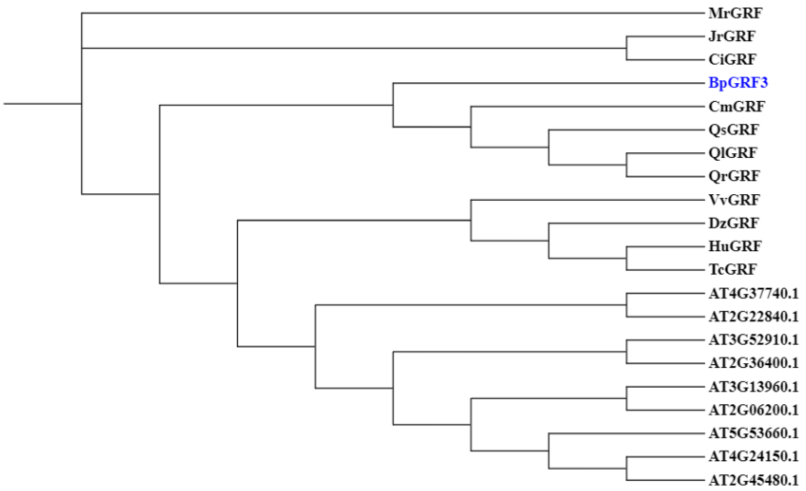

Supplemental Figure S3

Sequence and phylogenetic analysis of the BpGRF3 protein. (A) Multiple sequence alignment of BpGRF3 and GRF proteins from other species. The black line indicates the QLQ domain and WRC domain. (B) Phylogenetic analysis of BpGRF3 and GRF proteins from different plant species. The full names and accession numbers of the proteins analysed are listed in Supplementary Table S4.

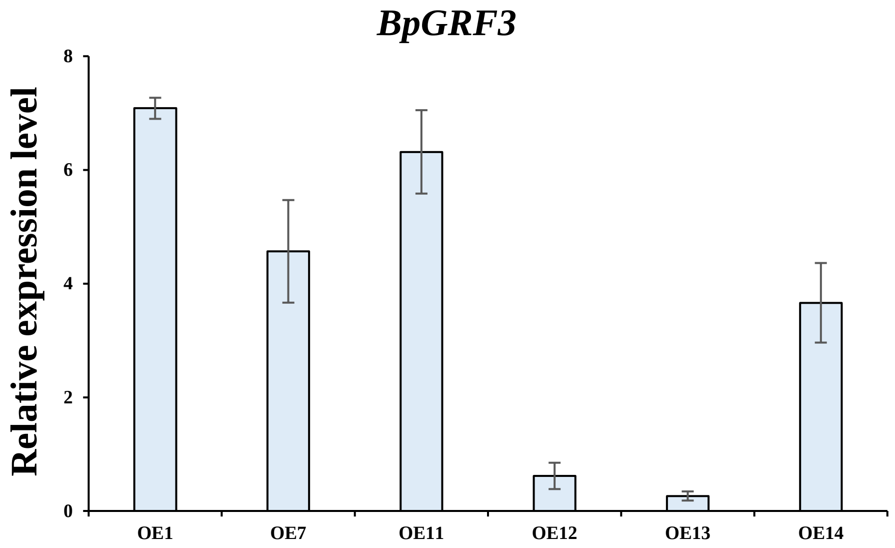

Supplemental Figure S4

Expression of *BpGRF3* in WT and transgenic birch. RT–qPCR analysis of the relative expression of *BpGRF3* in the *BpGRF3*-OE lines. All expression values were log2 transformed. The error bars represent the standard deviations (SD) of three biological replicates.

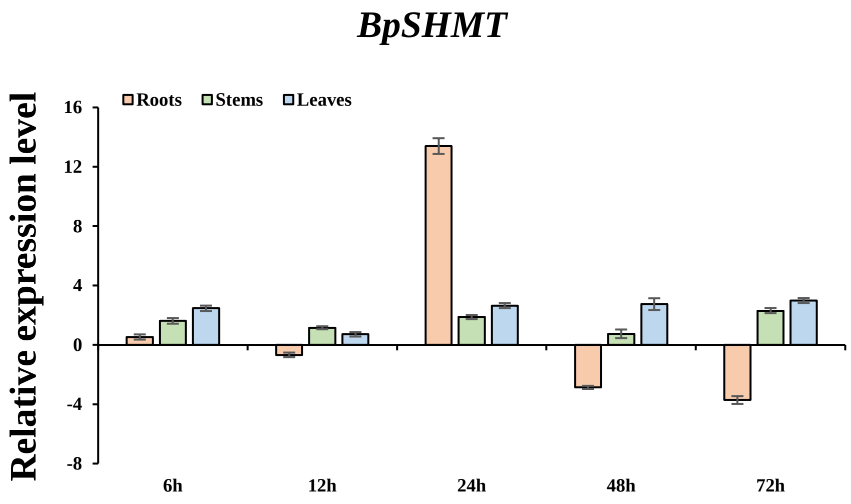

Supplemental Figure S5

Analysis of *BpSHMT* gene expression under salt stress. All expression values were log2 transformed. The error bars represent the standard deviations (SD) of three biological replicates.

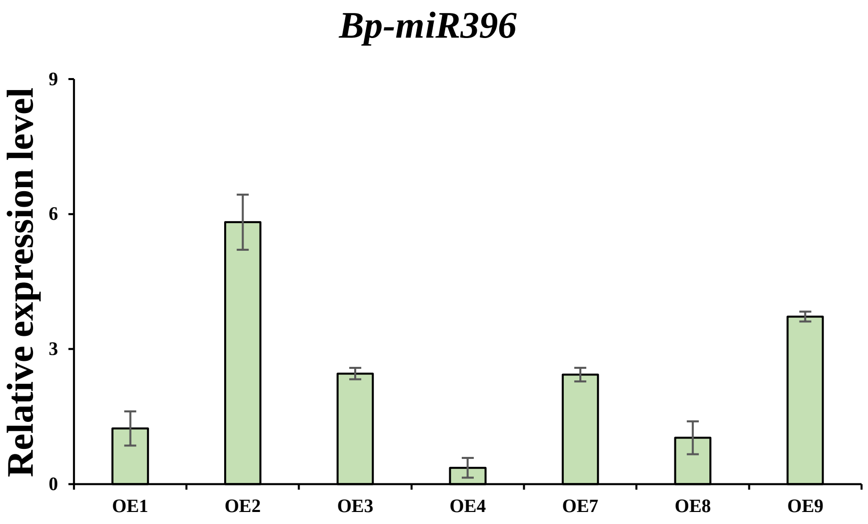

Supplemental Figure S6

Expression of *BpmiR396c* in WT and *BpmiR396c*-OE lines. All expression values were log2 transformed. The error bars represent the standard deviations (SD) of three biological replicates.
